# Establishment of SV40 Large T-Antigen-Immortalized Yak Rumen Fibroblast Cell Line and the Fibroblast Responses to Lipopolysaccharide

**DOI:** 10.3390/toxins15090537

**Published:** 2023-08-31

**Authors:** Junmei Wang, Ziqi Yue, Li Che, Hui Li, Rui Hu, Liyuan Shi, Xiaohong Zhang, Huawei Zou, Quanhui Peng, Yahui Jiang, Zhisheng Wang

**Affiliations:** Key Laboratory of Low Carbon Culture and Safety Production in Cattle in Sichuan, Animal Nutrition Institute, Sichuan Agricultural University, Chengdu 611130, China; junmeiwangsicau@163.com (J.W.); yzq947036981@163.com (Z.Y.); liche13453874699@163.com (L.C.); huilisicau@163.com (H.L.); ruitianhu@yeah.net (R.H.); shiliyuansicau@163.com (L.S.); zhangxiaohongsicau@163.com (X.Z.); zhwbabarla@126.com (H.Z.); pengquanhui@126.com (Q.P.); jiangyahui.ff@163.com (Y.J.)

**Keywords:** yak, cell culture, fibroblast cell line, lipopolysaccharide, extracellular matrix

## Abstract

The yak lives in harsh alpine environments and the rumen plays a crucial role in the digestive system. Rumen-associated cells have unique adaptations and functions. The yak rumen fibroblast cell line (SV40T-YFB) was immortalized by introducing simian virus 40 large T antigen (SV40T) by lentivirus-mediated transfection. Further, we have reported the effects of lipopolysaccharide (LPS) of different concentrations on cell proliferation, extracellular matrix (ECM), and proinflammatory mediators in SV40T-YFB. The results showed that the immortalized yak rumen fibroblast cell lines were identified as fibroblasts that presented oval nuclei, a fusiform shape, and positive vimentin and SV40T staining after stable passage. Chromosome karyotype analysis showed diploid characteristics of yak (n = 60). LPS at different concentrations inhibited cell viability in a dose-dependent manner. SV40T-YFB treated with LPS increased mRNA expression levels of matrix metalloproteinases (MMP-2 and MMP-9), inflammatory cytokines (TNF-α, IL-1β, IL-6), and urokinase-type plasminogen activator system components (uPA, uPAR). LPS inhibits the expression of tissue inhibitors of metalloproteinases (TIMP-1 and TIMP-2), plasminogen activator inhibitor-2 (PAI-2), fibronectin (FN), anti-inflammatory factor IL-10, and collagen I (COL I) in SV40T-YFB. Overall, these results suggest that LPS inhibits cell proliferation and induces ECM degradation and inflammatory response in SV40T-YFB.

## 1. Introduction

The yak (Bos grunniens) is a species of the bovine breed that lives on the Qinghai-Tibetan Plateau with an altitude of over 3000 m, especially in Gansu, Qinghai, and Tibet [1]. The yak is a precious genetic resource unique to China [2]. The yak holds significant value in the livelihoods of herders, contributing to various aspects such as the provision of food, hides, dung fuel, and serving as a source of transportation power. Additionally, the yak plays a crucial role in maintaining the balance of the natural ecosystems found in the Qinghai-Tibetan plateau [3]. Due to their special biological, economic, and distribution characteristics, the yak has been extensively studied in animal production in terms of nutrition, breeding, and genetics [4,5,6,7,8]. However, there are limited reports on these studies at the cellular level. So far, the cells related to the yak that have been isolated and cultured include yak germ cells, precursor adipocytes, rumen epithelial cells, fetal fibroblasts, mammary epithelial cells, etc. [9,10,11,12]. It is of great significance to construct an important yak population cell line to conserve its genetic resources from the perspective of our germplasm resource conservation.

Fibroblasts are the most common multifunctional cells in connective tissue and an important component of mesenchymal tissue [13]. It is known that one of the main functions of fibroblasts is the synthesis of collagen and other extracellular matrices (ECM), which plays an important role in the process of tissue and organ fibrosis [14]. Organ fibrosis is a condition characterized by the excessive accumulation of extracellular matrix (ECM) and myofibroblasts, and it is closely associated with chronic inflammatory diseases [15]. The signaling cascade leading to fibrosis during chronic inflammation, initiated by epithelial injury and leading to irreversible organ damage, is regulated by a range of inflammatory mediators [16]. The rumen is the most important organ of the digestive system of ruminants and an important part of the immune system of the organism [17]. The gastric wall consists of four layers: mucosa, submucosa, muscle, and epithelium, with the distribution of nerves, blood vessels, and lymphatic vessels [18]. Among them, fibroblasts are mainly distributed in the lamina propria, submucosa, and epithelium of the mucosal layer [19]. Fibroblasts have strong heterogeneity and are important for maintaining tissue homeostasis. Fibroblasts are important and valuable for the study of invasive metastasis, organ growth, angiogenesis, stromal remodeling, and immune regulation [20]. Ruminal bacteria are predominantly Gram-negative, and the release of LPS from bacterial lysis occurs in the rumen [21]. Under non-healthy conditions, such as ruminal acidosis, increased free ruminal LPS disrupts the rumen barrier function and causes increased permeability of the rumen epithelium, resulting in LPS translocating from the rumen into the blood circulation and activating an inflammatory response [22]. As a virulence factor, LPS can not only directly act on tissue cells to cause tissue destruction but also penetrate the epithelium into deep connective tissue to have a certain effect on fibroblasts [23,24].

The isolation and culture of fibroblasts are mainly used to study cell aging, cell damage by various foreign factors, malignant transformation of cells under in vitro conditions, as well as certain congenital metabolic abnormalities and enzyme defects [25,26,27,28,29]. In particular, some fibroblast cultures have been widely used in basic medicine and clinical medical research [30,31]. The primary cultured cells used for research can ensure that they are not affected by hemodynamics and other factors in vivo, and other influencing factors can be controlled by treatments such as drugs, providing a basis for studying various physiological and pathological processes [32,33,34,35]. Cell immortalization is expected to fundamentally solve the problem of the yak donor shortage and is currently a hot research topic internationally.

In the present study, we first described the establishment and characterization of the yak rumen fibroblast cell line. The identification of this cell line was carried out from the aspects of cell biological characteristics, cell function, cytogenetics, genetic markers, and other aspects to determine the successful establishment of the cell line. At the same time, we investigated the effect of LPS on the extracellular matrix of fibroblasts in rumen endotoxin diseases.

## 2. Results

### 2.1. Establishment of Primary and Yak Rumen Fibroblast Cell Lines

**Figure 1:**
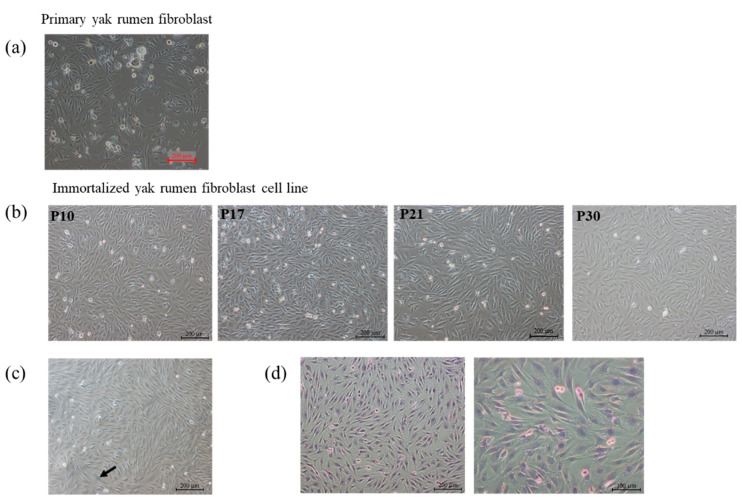
Morphology of primary yak rumen fibroblasts and establishment of fibroblast cell lines. (**a**) Primary cells (×100). (**b**) Cell morphology of yak rumen fibroblast cell lines with different generations of passage (×100). (**c**) Cellular senescence SA-β-gal staining (×100). (**d**) Hematoxylin–eosin (HE) staining (×100, ×200).

The primary fibroblasts cultured from the yak rumen papilla tissue block are shown in Figure 1a. The fibroblastic cells, which exhibited morphological features such as spindle-like, bipolar, or multipolar shapes, elongated morphology, and adhesive growth, were documented through phase contrast microscopy. At the same time, it was also found that some cells showed the form of senescence, with the characteristic of cell mass enlargement and severe vacuolation, which were also the disadvantages of cultivating primary cells. The proliferation rate of primary cells decreased dramatically with the increase in passage times. To obtain stable passage and high proliferation efficiency of cells, we first report that an immortalized yak rumen fibroblast cell line (SV40T-YFB) was established through infection with lentivirus expressing SV40 large T antigen (Figure 1b). The immortalized yak rumen fibroblast cell line could be subcultured for at least 30 passages with stable proliferation ability (Figure 1b). In addition, no dramatic shape differences were found when the fibroblast cells at earlier and later passages were compared, indicating this newly established fibroblast cell line is stable. The 30th generation cells were taken to detect the senescence state of the cells. The senescent cells are commonly characterized by an enlargement in cell volume and upregulated enzymatic activity of β-galactosidase at pH 6.0. Subsequent staining of these cells results in the generation of a dark blue product. As shown in the Figure 1c, there was no significant amount of dark blue product staining throughout the field of view in the rumen immortalized yak cells under the microscope. The minimal presence of cells displaying a light blue color, as indicated by the arrows in the accompanying image, indicates a significantly lower level of β-galactosidase expression in normal cells. In contrast, aging cells exhibit a more intense cellular coloration following staining. The results indicated that the cells had strong proliferation ability and no obvious senescence (Figure 1c). Hematoxylin–eosin (HE) staining also showed that the morphology of the whole cells was visible. Hematoxylin was used to stain the nucleus in a blue-purple color, while eosin was employed to stain the cytoplasm in a pink hue (Figure 1d).

### 2.2. Transfection Efficiency of SV40T Gene, Immunofluorescence Identification, and Growth Curve in Yak Rumen Fibroblast Cell Lines

**Figure 2:**
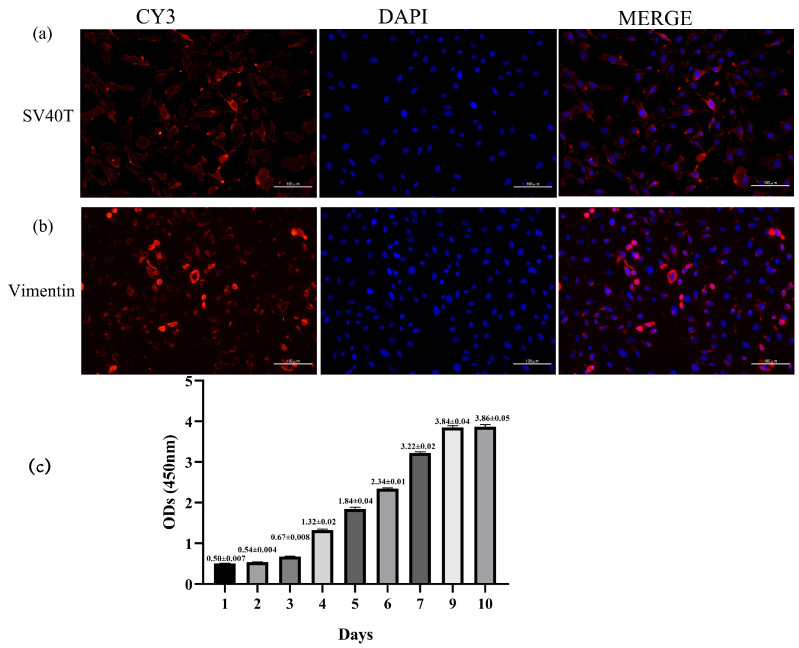
Identification of yak rumen fibroblast cell line. (**a**) SV40 large T antigen (SV40T) transfection was observed under an immunofluorescence microscope (×200). (**b**) Cells exhibited positive expression for intracellular vimentin merged with DAPI, and DAPI showed a prominent nucleus (×200). (**c**) Growth curve of cultured yak rumen fibroblast cells (n = 5). Results are presented as mean ± SEM.

Primary yak rumen fibroblasts were infected with lentivirus expressing SV40 large T antigen, which was produced by co-transfecting lentiviral vector (pGMLV-SV40T-PURO) and packaging vectors. The presence of the SV40 large T antigen was validated by immunofluorescence analyses. The results showed that cells expressed SV40 large T antigen (Figure 2a). Vimentin is an intermediate filament that can indicate the mesenchymal origin of fibroblast cells. We used vimentin as a dermal fibroblast marker and found strong immunoreactivity of yak rumen fibroblast cells to vimentin (Figure 2b). Cell proliferation was assessed using the CCK8 assay, and the growth curve was depicted in Figure 2c. The growth curve exhibited a characteristic “S” shape, indicating that the cells underwent incubation, logarithmic growth, and plateau stages, in accordance with the typical growth pattern of cells. These results indicated that the established yak rumen fibroblast lines were consistent with the morphological characteristics and proliferation capacity of normal fibroblasts.

### 2.3. Specific Analysis and Identification of Cell Species

**Figure 3:**
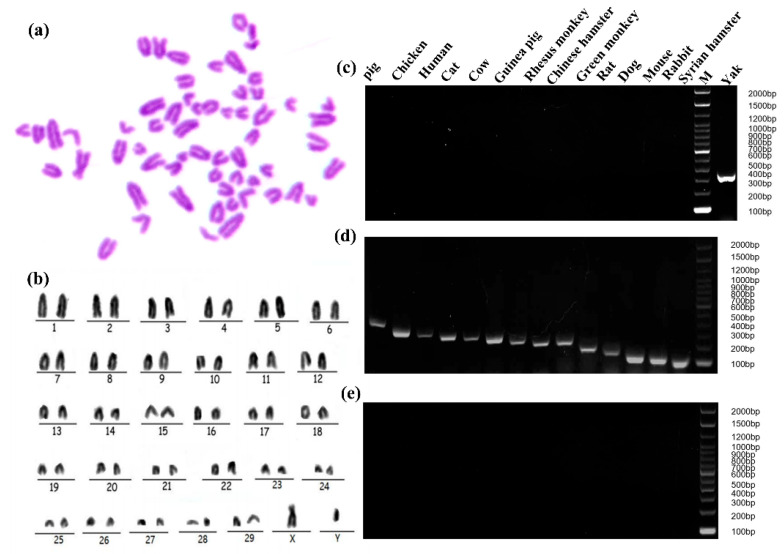
Genetic characteristic analysis of immortalized yak rumen fibroblast cell lines. (**a**) Karyotype analysis of yak rumen fibroblast cell line (SV40T-YFB). (**b**) Chromosomal rearrangements of SV40T-YFB. (**c**) PCR product of SV40T-YFB. (**d**) PCR products from 14 common cell species (pig, chicken, human, cat, cow, guinea pig, rhesus monkey, Chinese hamster, green monkey, rat, dog, mouse, rabbit, Syrian hamster). Positive control, template is species DNA. (**e**) The PCR results of 14 species-specific fragments (negative control, the template is sterile ultrapure water).

Chromosome karyotype analysis and species identification methods were used to prove the origin of cell lines from cytogenetics characteristics. The G-banding technique was used to color different regions of the chromosomes by a special staining method so that the chromosomes showed light and dark banding under light microscopy (Figure 3a). The chromosomes were analyzed, compared, sorted, and numbered according to the characteristics of chromosome length, centromere location, the ratio of long and short arms, and the presence or absence of a follower. The findings revealed that cell line exhibited a diploid karyotype, constituting 100% of the observed karyotypes. This observation demonstrated the characteristic and representative diploid nature of the yak species (2n = 60) without any instances of polyploidy-induced mutations. The chromosome arrangement includes 29 pairs of autosomes and one pair of sex chromosomes (Figure 3b). Positive controls were observed by agarose electrophoresis of PCR products from 14 common cell species (pig, chicken, human, cat, cow, guinea pig, rhesus monkey, Chinese hamster, green monkey, rat, dog, mouse, rabbit, Syrian hamster) (Figure 3d). The immortalized yak rumen fibroblast cell line (SV40T-YFB) only showed specific fragments under yak primer amplification, and no cross-contamination of other species cells was found in the cells (Figure 3c).

### 2.4. Effect of Lipopolysaccharide on the Cell Viability of Yak Rumen Fibroblasts

**Figure 4:**
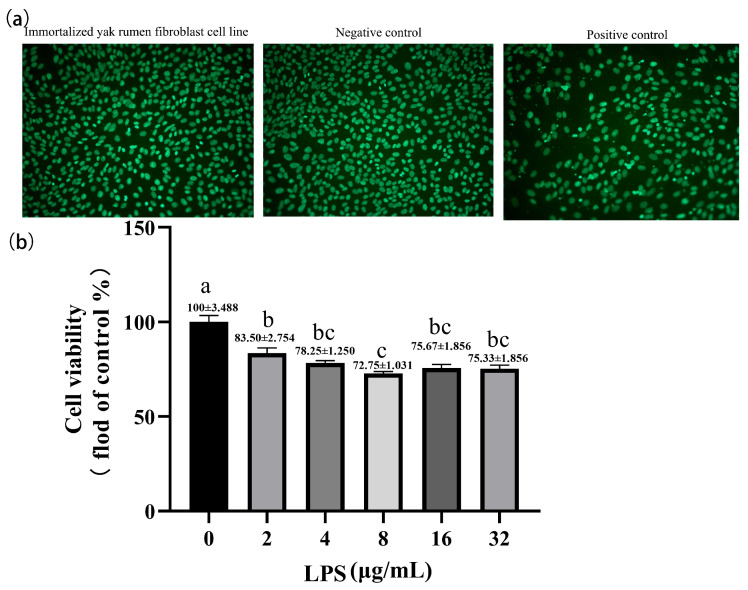
Effects of LPS at different concentrations on cell viability in yak rumen fibroblasts (SV40T-YFB). (**a**) DNA staining method for detecting mycoplasma contamination. (**b**) Cell viability was measured after treating the immortalized cell lines with LPS at different concentrations (2, 4, 8, 16, and 32 µg/mL) for 24 h (n = 6). Data are expressed with one-way ANOVA as means ± SEM. The letters above the bars indicate statistically significant groups. Different letters indicate significantly different values among treatments at *p* < 0.05. Columns with the same letter indicate lines that were not different (*p* > 0.05).

We inspected the cell cultures repeatedly under the light microscope for potential microorganism contamination and did not find any rapid-onset turbidity, culture medium color change, or moving granules between cells, which would indicate contamination of bacteria before applying this cell line to the experiment. Moreover, a DNA fluorescence staining technique was performed to test cultures for mycoplasma contamination. Expectedly, the results of fluorescence staining of mycoplasma were negative suggesting that the cell cultures were free of microorganism contamination (Figure 4a). We measured the cell viability of SV40T-YFB after treatment of LPS for 24 h. The cell growth was significantly inhibited by increasing LPS concentration in a dose-dependent manner (*p* < 0.001; Figure 4b).

### 2.5. Effects of LPS on the Expression of ECM-Degradation-Related Genes in Yak Rumen Fibroblasts

**Figure 5:**
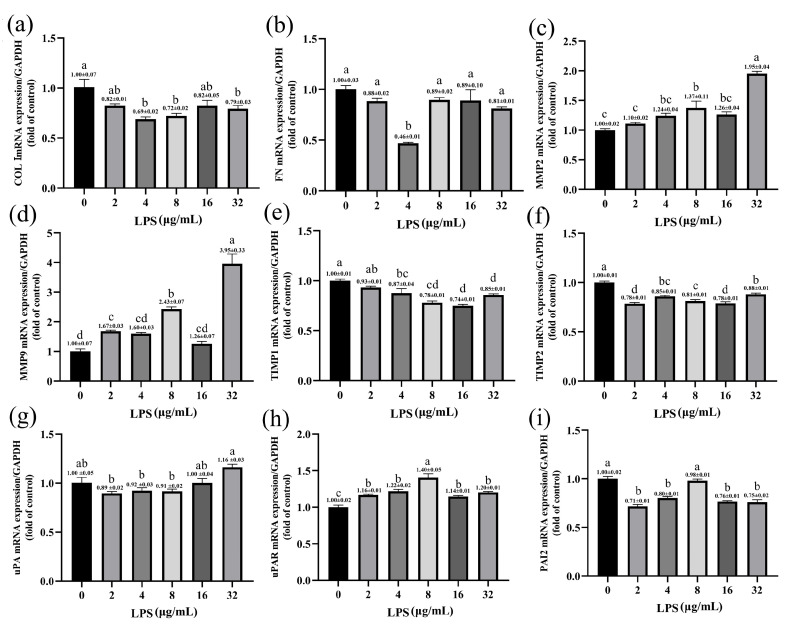
Effects of LPS on the expression of ECM-degradation-related genes in yak rumen fibroblasts (SV40T-YFB). SV40T-YFB cells were cultured without stimulation and treated with various concentrations of LPS (2, 4, 8, 16, and 32 μg/mL) for 24 h. Subsequently, the mRNA levels of COL Ι (**a**), FN (**b**), MMP-2 (**c**), MMP-9 (**d**), TIMP-1 (**e**), TIMP-2 (**f**), uPA (**g**), uPAR (**h**), and PAI-2 (**i**) were determined using quantitative polymerase chain reaction (qPCR) (n = 3). Data are expressed with one-way ANOVA as means ± SEM. The letters above the bars indicate statistically significant groups. Different letters indicate significantly different values among treatments at *p* < 0.05. Columns with the same letter indicate lines that were not different (*p* > 0.05).

The qPCR was performed to quantitatively measure the expression of ECM genes in SV40T-YFB after LPS treatment with different concentrations. LPS inhibited the expression of collagen Ι (COL Ι) and fibronectin1 (FN). The 4, 8, and 32 μg/mL LPS significantly inhibited the expression of the COL Ι gene (*p* < 0.001; *p* < 0.01; *p* < 0.05; Figure 5a). LPS (4 μg/mL) significantly affected the mRNA expression of FN (*p* < 0.001; Figure 5b). Compared with the control group, stimulation of the cells with LPS significantly increased matrix metalloproteinase (MMP-2 and MMP-9) mRNA levels at a relatively high dose, especially those treated with 32 μg/mL LPS (*p* < 0.001; Figure 5c,d). No significant changes were observed in the MMP-2 mRNA level when the concentration of LPS was ≤ 4 μg/mL (*p* > 0.05; Figure 5c). Correspondingly, the expression of the MMP-9 gene was significantly increased with the increase in LPS concentration (Figure 5d). However, mRNA levels of MMP-2 and MMP-9 did not change significantly in the yak rumen fibroblasts treated with 16 μg/mL LPS (*p* > 0.05). Based on increased MMPs mRNA expression, we next examined mRNA expression of the tissue inhibitors of metalloproteinases (TIMPs) in yak rumen fibroblasts. The expression of TIMP-1 and TIMP-2 genes decreased in the treatment of LPS (Figure 5e,f). The down-regulated expression of the TIMP-1 gene showed a dose-dependent trend with LPS concentration. We examined the effect of different concentrations of LPS on the constitutive expression of three urokinase-type plasminogen activator (uPA) system components: uPA, uPA receptor (uPAR), and plasminogen activator inhibitor-2 (PAI-2) in yak rumen fibroblasts. The data indicates that LPS induces a higher expression of uPAR mRNA compared to the control group, reaching its peak at 8 μg/mL LPS concentration (*p* < 0.001; Figure 5h). Nonsignificant effects were observed on uPA expression with different concentrations of LPS in yak rumen fibroblasts (*p* > 0.05; Figure 5g). The expression of PAI-2 mRNA was decreased in different concentrations of LPS-treated fibroblasts except that 8 μg/mL LPS treated cells had no significant effect on the expression of PAI-2 gene (*p* < 0.001; *p* > 0.05; Figure 5i).

### 2.6. Effects of LPS on the Expression of Inflammatory-Response-Related Genes in Yak Rumen Fibroblasts

**Figure 6:**
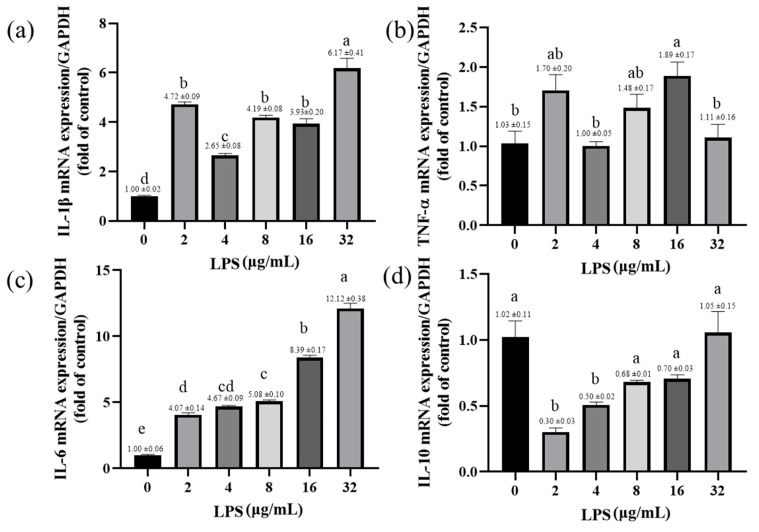
Effects of LPS on the expression of inflammatory-response-related genes in yak rumen fibroblasts (SV40T-YFB). SV40T-YFB cells were cultured under unstimulated conditions and the LPS at 2, 4, 8, 16, and 32 μg/mL were treated for 24 h. After treatments, the IL-1β (**a**), TNF-α (**b**), IL-6 (**c**), and IL-10 (**d**) mRNA levels were determined by qPCR, respectively (n = 3). Data are expressed with one-way ANOVA as means ± SEM. The letters above the bars indicate statistically significant groups. Different letters indicate significantly different values among treatments at *p* < 0.05. Columns with the same letter indicate lines that were not different (*p* > 0.05).

LPS increases the expression of inflammatory factors in yak rumen fibroblasts. The expression of IL-1β was significantly increased in rumen fibroblasts exposed to different LPSs (*p* < 0.001; Figure 6a). The expression of TNF-α mRNA was significantly increased in the yak rumen fibroblasts exposed to 16 μg/mL LPS (*p* < 0.05; Figure 6b). Similarly, LPS treatment with different concentrations significantly increased the expression of IL-6 gene in SV40T-YFB in a dose-dependent manner (*p* < 0.001; Figure 6c). The expression of IL-10 mRNA was significantly inhibited by LPS exposure to yak rumen fibroblasts of 2 and 4 μg/mL (*p* < 0.001; *p* < 0.01; Figure 6d). LPS significantly increases the expression of pro-inflammatory cytokines and inhibits the expression of anti-inflammatory factors, thereby affecting the inflammatory response.

## 3. Discussion

Immortalized cells are defined as the process in which diploid cells cultured in vitro escape from the crisis of proliferation and senescence spontaneously or under the influence of external factors and have infinite proliferation capacity. However, the probability of immortalization of diploid cells is very small under spontaneous conditions [36,37]. To obtain the characteristics of normal primary cells that can be cultured and proliferated in vitro, retrovirus vectors were constructed to transfer the immortalized genes into the cells, so that the primary cells could maintain their physiological and biochemical characteristics even after immortalization [38,39]. SV40T is currently the target gene commonly used for immortalized cell transfection of cells internationally in vitro [40]. SV40T gene can affect telomere length and activate telomerase activity [41]. Telomeres are composed of guanine-rich DNA repeats and related proteins located at the 3′ end of eukaryotic chromosomes [42]. Telomerase activation and telomere length stability are closely related to cell replication senescence and immortalization [43]. We successfully immortalized yak rumen fibroblasts by stably transfecting primary cells with the SV40T gene mediated by lentivirus. Fibroblasts are spindle-shaped or flat and star-shaped, with large cells and nuclei and protruding contours, which makes them different from other structural cells, such as epithelial cells and endothelial cells [44]. The morphology of the yak rumen fibroblasts in this study was consistent with the results of previous studies. The detection of fibroblast marker-specific protein, immortalized gene SV40T, and cytogenetics characteristics confirmed that the function of the cell line was stable and could be used for subsequent research.

The rumen wall consists of serosa, muscularis, mucosa and submucosa. Fibroblasts are the main cellular component of the sparse connective tissue present in the submucosal layer [45]. Fibroblasts are usually considered “immune neutral” cells to construct and reshape ECM and play a role in structural support and maintenance of tissue integrity [44,46]. Fibroblasts activate pro-inflammatory signaling pathways by pathologically stimulating injury-associated molecular patterns and pathogen-associated molecular patterns [47,48,49]. LPS is a highly proinflammatory endotoxin in the circulatory system, past studies have raised concerns regarding ruminal LPS contribution to the pathogenesis of ruminal acidosis [21]. In this study, LPS inhibited the proliferation of yak rumen fibroblasts, and cell viability was severely affected with increasing concentrations. LPS stimulates the immune response of fibroblasts and activates the NF-κB pathway inducing the production of various pro-inflammatory factors such as interleukin-1, IL-6, and TNF-α, leading to an inflammatory response [50]. In the current study, cultured yak rumen fibroblasts were exposed to LPS in a dose-dependent manner to further characterize the proinflammatory response. In experiment, expression of interleukin-1, IL-6, and TNF-α were upregulated following exposure to LPS, confirming that yak rumen fibroblasts are capable of expressing proinflammatory cytokine genes. In this experiment, a clear dose-dependent effect on gene expression levels was not observed, which may be attributed to the ability of LPS to activate multiple signaling pathways, including toll-like receptors (TLRs) and cytokine receptors, exhibiting cross-regulatory characteristics [51,52]. Therefore, at different doses, different signaling pathways may be activated, leading to a more complex pattern of gene expression modulation, beyond the influence of dosage alone.

In pathological processes such as inflammation, tissue damage, and repair, proteases play an important role in the degradation of ECM, which mainly contains collagen, non-collagen, elastin, proteoglycan, and aminoglycan components [53]. Studies have shown that the ECM content of elastin, laminin, and fibrin decreased significantly after tissue damage [54]. uPA is an important serine protease that binds to the uPA receptor (uPAR) on the surface of cell membranes and is activated to activate a series of proteases, such as fibrinogenase, thereby degrading ECM [55]. Fibrinolytic enzymes are trypsin-like proteases that not only degrade a variety of extracellular matrices such as fibrin but also activate a variety of proteases such as matrix metalloproteinases (MMPs) [56,57]. Thus, uPA plays a key role in initiating and promoting the degradation of the entire ECM. Inhibition of MMP proteolytic activity or the upregulation of TIMPs were considered to be responsible for the accumulation of ECM and fibrosis [58,59]. High expression of uPA and uPA receptors in the uPA-fibrinolytic enzyme system in gingival fibroblasts was found to lead to periodontal tissue damage [60]. In the process of human skin and pulmonary fibrosis, the uPAR of fibroblasts was knocked out to produce more ECM, but when the uPA inhibitor plasminogen activator inhibitor-1 (PAI-1) was knocked out, collagen secretion was inhibited and fibroblast proliferation and migration were significantly suppressed [61,62,63]. The accumulation of ECM and fibrosis is believed to be attributed to either the inhibition of MMPs proteolytic activity or the upregulation of TIMPs [15]. LPS also activates the uPA-plasminase system, which contributes to the degradation of the ECM in fibroblasts [63,64]. Organ fibrosis is a disease characterized by excessive production of ECM excessive [65], and an abnormal inflammatory response can further contribute to ECM overproduction and deposition, leading to fibrosis [24]. Previous studies have provided evidence for the involvement of matrix metalloproteinase (MMP) gene expression and activity in ECM catabolism, as well as the role of the urokinase-type plasminogen activator (uPA) system [66]. In our study, we found that LPS affects the expression of ECM-related genes, leading to impaired functions of fibroblasts. However, with increasing LPS concentration, gene expression did not show a clear dose-dependent effect. This phenomenon may be attributed to the dynamic balance and interactions among components of the ECM. Molecules like MMPs secreted by cells and tissues degrade ECM fibers and crosslinks, contributing to the dynamic equilibrium of ECM [67]. While MMPs generally weaken ECM, cells also synthesize collagen (especially types I and III) to create new fibers and form crosslinks between existing ones, maintaining ECM structure and function [68]. These biochemical processes continuously sculpt ECM, ensuring its dynamic equilibrium [69]. However, to the best of our knowledge, our study represents the first comprehensive investigation of the association between MMPs/TIMPs and the uPA system in yak rumen fibrosis at the gene level in in vitro cell experiments, providing a theoretical basis for further exploration of its regulatory mechanisms. By examining LPS-induced yak rumen fibroblasts, we speculate that the degradation of ECM by MMPs/TIMPs, along with the involvement of the uPA system and inflammatory responses, may contribute to the development of yak rumen fibroblast damage, ultimately leading to yak rumen fibrosis. Additionally, we indirectly demonstrated the normal functionality of the fibroblast cell line established, enabling basic experimental research.

## 4. Conclusions

In summary, we isolated and cultured yak rumen fibroblasts for the first time and established a yak rumen fibroblast cell line with normal morphology and function. This cell line can stably passage for more than 30 generations without any changes in chromosome karyotype. Our study revealed the activation of the uPA system and an inflammatory response in the yak rumen fibroblasts treated with different concentrations of LPS. This activation potentially influences the metabolism of ECM in yak rumen fibroblasts and may contribute to the development of yak rumen fibrosis. These findings highlight the involvement of the inflammatory response, MMPs/TIMPs, and the uPA system as potential mechanisms underlying yak rumen fibrosis. Future studies will aim to validate and further elucidate these mechanisms, which could potentially pave the way for the development of novel therapies for yak rumen fibrosis targeting the inflammatory response, MMPs/TIMPs, and the uPA system.

## 5. Materials and Methods

### 5.1. Isolation and Cultivation of Primary Yak Rumen Fibroblasts

The rumen epithelial tissue was obtained from a yak growing on the Qinghai Tibet Plateau at an abattoir. Immediately after slaughter, tissue samples (about 5 cm^2^ in size) were excised from the rumen epithelial and cleaned with phosphate-buffered saline (PBS; Solarbio, Beijing, China) with 400 U/mL penicillin and 400 μg/mL streptomycin, then collected into separate tubes containing Roswell Park Memorial Institute (RPMI; Gibco, Carlsbad, CA, USA) 1640 culture medium with 200 U/mL penicillin and 200 μg/mL streptomycin and transported to the laboratory on ice within 6 h. Following aseptic techniques, the rumen epithelial papilla was rinsed and finely diced into 1 mm^2^ size fragments using sterile surgical scissors. Subsequently, the tissue pieces were subjected to three washes with PBS supplemented with 100 U/mL penicillin and 100 μg/mL streptomycin. After that, these pieces were immersed in the RPMI 1640 medium containing 10% fetal bovine serum (FBS; Cell-Box, HK, China), 10 ng/mL basic fibroblast growth factor (bFGF; R&D Systems, Minneapolis, MN, USA) and antibiotics (100 U/mL penicillin, 100 μg/mL streptomycin; Solarbio, Beijing, China) for 5 min. Then, the tissue was seeded on the bottom of 25 cm^2^ tissue culture flasks (Corning, NY, USA) at 37 °C with saturated humidity and 5% CO_2_ for 5 h. Following the addition of RPMI 1640 medium, the tissue pieces were allowed to adhere spontaneously to the surface of the flask. Once the cells reached 80–90% confluence, they were digested using a solution of 0.25% trypsin and 0.04% EDTA (Beyotime, Shanghai, China). Subsequently, the cells were subcultured at an approximate ratio of 1:3. The cells were then purified and expanded through serial passaging.

### 5.2. The Establishment of Yak Rumen Fibroblast Cell Line

The 1 × 10^4^ cells/mL density was seeded in 96 well plates. SV40 large T-antigen lentivirus (VectorBuilder, Guangzhou, China) was prepared with 8 mg/L polybrene (Sigma-Aldrich, St. Louis, MO, USA), and RPMI 1640 culture medium with multiple infection numbers (MOIs) of 10, 20, 30, 40, 50, 60, 70, 80, 90, 100 was added into each well after the primary cells were cultured to 50% cell density. The cells were infected overnight with lentivirus. Then, the virus supernatant was discarded and rinsed 3 times with PBS, and fresh medium was added for 4 days of culture. The puromycin (Sigma-Aldrich, St. Louis, MO, USA) with concentration gradients of 0, 0.5, 1, 2, 4, 5, 6, and 7 μg/mL to screen the surviving cell lines when the cell density grew to 20–30%. The cell growth was observed and recorded, and the puromycin medium was changed every 2 days for further screening. After 96 h of continuous culture, the surviving cells were rumen fibroblast cell lines of the yak. RPMI 1640 complete medium containing 10% fetal bovine serum,100 U/mL penicillin, 100 U/mL streptomycin, and 10 ng/mL basic fibroblast growth factor were used to routinely culture the cell lines after cell growth and fusion.

### 5.3. Senescence-Associated β-Galactosidase (SA-β-Gal) and Hematoxylin–Eosin (HE) Staining 

Yak rumen fibroblasts of the 30th generation were cultured in 24-well plates (1 × 10^4^ cells/well). The cells were washed with PBS and fixed in 4% paraformaldehyde at room temperature when the cell density grew to 80%. SA-β-gal staining was performed using a senescence β-galactosidase staining kit (Beyotime, Shanghai, China) according to the manufacturer’s instructions. The number of senescent cells that produce the dark blue product β-galactosidase catalyzed by senescence-specific β-galactosidase using X-Gal as a substrate was observed under a light microscope (magnification, ×100). For HE staining (Beyotime, Shanghai, China), the hematoxylin staining solution was used to stain the nucleus, and the eosin staining solution to stain the cytoplasm after cell fixation. Finally, the cell staining state was observed under a microscope.

### 5.4. Cell Viability

Yak rumen fibroblasts from the 10th generation were seeded in 96-well plates at a density of 1 × 10^4^ cells/mL. Each treatment group was performed in six replicates, and the viability of one group of cells was assessed daily. Cell viability was measured using a Cell Counting Kit-8 (CCK-8; AbMole, Shanghai, China). To conduct the assay, 10 µL of CCK-8 reagent was added to each well following the instructions of manufacturer [70]. The absorbance was measured at 450 nm wavelength in a microplate reader (SpectraMax M2, USA) after 4 h of incubation at 37 °C. Using the incubation time as the abscissa and the average OD value as the ordinate, the cell growth curve was drawn. Yak rumen fibroblasts were seeded in 96-well plates at a density of 1 × 10^4^ cells/mL and challenged with LPS (Solarbio, Beijing, China) at different concentrations (0, 2, 4, 8, 16, and 32 μg/mL) for 24 h. In this study, we selected a range of LPS concentrations to reflect the reported concentrations of rumen LPS in the literature and the experimental concentrations that affect the functionality of fibroblast cells in vitro [71,72]. The CCK-8 was used to detect the effect of lipopolysaccharide on cell viability.

### 5.5. Immunofluorescence Identification

Yak rumen fibroblasts of the 5th generation were seeded in 6-well plates at a density of 1 × 10^4^ cells/mL. The immunofluorescence detection method was according to the previous literature [10]. In brief, the cells were fixed with 4% paraformaldehyde at room temperature for 15 min when the cells grew to 90% confluence. After the cells were washed with PBS 3 times, 0.1% Triton X-100 was used to permeate the cell membranes for 10 min. Then, the cells were blocked with 5% goat serum (Solarbio, Beijing, China) at room temperature for 30 min. The immortalized cell lines were incubated with anti-SV40T-antigen antibody and anti-vimentin antibody (1:50; Abcam, Cambridge, MA, USA), respectively, at room temperature for 4 h and then cocultured with CY3-conjugated goat anti-rabbit IgG antibodies (1:3000; Thermo Fisher Scientific, Waltham, MA, USA) at room temperature for 1 h. Cells at each step were washed 3 times with PBS. The cell nuclei were stained with 4′,6-diamidino-2-phenylindole (DAPI; Solarbio, Beijing, China) for 5 min. A fluorescence microscope was used for observation (Nikon, Tokyo, Japan).

### 5.6. Karyotype Analysis

The number of chromosomes in the yak rumen fibroblast cell line was determined following standard methods [73,74]. To prepare for chromosomal analysis, colcemid (Solarbio, Beijing, China) was added to the cells at a final concentration of 0.6 μg/mL and incubated at 37 °C for 5 h. Cell division was halted at the intermediate phase after 2–4 h of incubation at 37 °C in a 5% CO_2_ incubator. The cell suspension was collected and treated with 8 mL of 0.075 mol/L potassium chloride for 15 min at room temperature (RT). Subsequently, the cells were fixed three times with a freshly prepared ice-cold solution of acetic acid-methanol (1:3, vol/vol). The suspension was then dropped onto clean slides that were either dipped in ice water or air-dried, blown away, heated, and air-dried. The chromosomes were stained with a Giemsa stock solution (diluted with pH 6.8 phosphoric acid buffer in a 9:1 ratio) for 15–20 min, followed by washing with water and air drying. Metaphase cells with well-dispersed chromosomes were selected using a low-power microscope, and further observation was performed using an oil microscope.

### 5.7. Cell Species Identification

The immortalized yak rumen fibroblast cell line (SV40T-YFB) at passage 30 was sent to the China Center for Type Culture Collection (CCTCC) for preservation (CCTCC NO. C2021253). The cell lines were characterized by CCTCC using species identification. The DNA of the cells was extracted for polymerase chain reaction (PCR) detection. The 14 common cell species of primers specific (pig, chicken, human, cat, cow, guinea pig, rhesus monkey, Chinese hamster, green monkey, rat, dog, mouse, rabbit, Syrian hamster) were selected for PCR, and the PCR products were observed by agarose electrophoresis to determine the species origin of the cells and to determine whether there was cross-contamination of the cells. The PCR primers are not publicly available.

### 5.8. DNA Fluorescence Staining

According to the ATCC animal cell line quality control method in the United States, DNA fluorescence staining was used to detect mycoplasma [75]. Vero cells were seeded in 6-well plates at a density of 1 × 10^4^ cells/mL. The antibiotic-free medium (RPMI 1640 complete medium containing 10% fetal bovine serum) was used to culture yak rumen fibroblasts for one day and then 1 mL of culture medium was used to culture Vero cells for 5 days. Infection of Vero cells with B6YH4 (hybridoma cell, mycoplasma positive) was used as a positive control, while the negative control was obtained from a fresh culture medium of Vero cells. After the cells were washed with PBS 3 times, the cells were fixed with 4% paraformaldehyde at room temperature for 10 min. The fluorescent dye working solution was added to the stain for 10 min. A fluorescence microscope was used for observation (Nikon, Tokyo, Japan). Each step involved three washes with PBS.

### 5.9. RNA Was Extracted and Analyzed Using Quantitative Reverse Transcription Polymerase Chain Reaction (qRT–PCR) for Gene Expression

Yak rumen fibroblasts were seeded in 6-well plates at a density of 1 × 10^5^ cells/mL, and challenged with LPS (Solarbio, Beijing, China) at different concentrations (0, 2, 4, 8, 16 and 32 μg/mL). Then, these cells were washed with PBS. Cellular total RNA was extracted using a total RNA extraction kit (Yeasen Biotechnology, Shanghai, China). To determine gene expression, qPCR was performed following previously reported methods [76,77]. Reverse transcription was carried out using the SweScript All-in-One Blue RT SuperMix for qPCR (One-Step gDNA Remover) (Servicebio Biotechnology, Shanghai, China). A reverse transcription reaction volume of 20 μL was used, with 1 μg of total RNA. For qRT-PCR, the 2× Universal Blue SYBR Green qPCR Master Mix kit (Servicebio Biotechnology, Shanghai, China) was employed. The qPCR system (QuantStudio 5, Foster City, CA, USA) involved an initial denaturation step at 95 °C for 30 s, followed by 40 cycles of denaturation at 95 °C for 15 s and annealing/extension at 60 °C for 30 s. The internal control for normalization was glyceraldehyde-3-phosphate dehydrogenase (GAPDH), and the primer sequences used for the quantitative assays are provided in Table 1. The relative fold change in mRNA expression was determined using the 2^−ΔΔCt^ method [78].

### 5.10. Data Analysis

The experiment results are expressed as the mean ± standard error (mean ± SEM), and statistical analysis and mapping were performed using GraphPad Prism version 8.0 software. Differences between groups were compared by one-way ANOVA/Tukey’s multiple comparison tests. *p* < 0.05 was considered statistically significant.

## Figures and Tables

**Table 1:** The Gene Sequence Information.

Gene	Forward (5′→3′)	Reverse (5′→3′)	Accessin No.
MMP2	GATAACCTGGATGCTGTGGTGGAC	TGCTTCCGAACTTCACGCTCTTC	XM_005890934
MMP9	CTGGCTTGCTGCTCTGCTGTC	CTGGCTTGCTGCTCTGCTGTC	XM_005901514
uPA	CAGGTCACCAACGCCGAGAAC	GATGAGGCTGCCACCACACAAG	XM_005901514
uPAR	GCTTCAAAACCTGCCACCAAACG	GTCCTTTAGTGCCTGTCGCTTCC	XM_005899359
TIMP-1	CCAGAACCGCAGTGAGGAGTTTC	CAGCAGCATAGGTCTTGGTGAATCC	XM_005899359
TIMP-2	GCTGGACATTGGAGGAAAGAAGGAG	CAGGGCACGATGAAGTCACAGAG	XM_014483144
PAI-2	AGGCGGTGGACTTCCTAGAACG	GTAGACAGCATTCACCAGGACCATC	XM_005906498
COL-Ι	CCGAGGGCAACAGCAGATTCAC	TCAAGGATAGGCAGGCGAGATGG	XM_005909695
FN	TGGCGAGTGGAAGTGTGAGAGG	ATACGGAGGCGGCTGAGGATG	XM_014482895
IL-1β	CTCCGACGAGTTTCTGTGTGACG	GAGAGGAGGTGGAGAGCCTTCAG	NM_174093.1
IL-6	CACTGACCTGCTGGAGAAGATGC	CCGAATAGCTCTCAGGCTGAACTG	XM_005901249.2
TNF-α	TGAAGGAAGAGGAGAGGCTCATCG	GTGGTCATCGGAGTTGCTGGTG	XM_005904178.1
IL-10	GAACCACGGGCCTGACATCAAG	CTTCTCCACCGCCTTGCTCTTG	XM_005891650
GAPDH	GGGTCATCATCTCTGCACCT	GGTCATAAGTCCCTCCACGA	XM_014482068.1

## Data Availability

The datasets used and/or analyzed during the current study are available from the corresponding author upon reasonable request.
